# Analyze the Diversity and Function of Immune Cells in the Tumor Microenvironment From the Perspective of Single‐Cell RNA Sequencing

**DOI:** 10.1002/cam4.70622

**Published:** 2025-03-10

**Authors:** Lujuan Ma, Yu Luan, Lin Lu

**Affiliations:** ^1^ Department of Medical Oncology, Guangzhou First People's Hospital, School of Medicine South China University of Technology Guangzhou Guangdong China

**Keywords:** cancer, immune cells, single‐cell sequencing, tumor microenvironment

## Abstract

**Background:**

Cancer development is closely associated with complex alterations in the tumor microenvironment (TME). Among these, immune cells within the TME play a huge role in personalized tumor diagnosis and treatment.

**Objectives:**

This review aims to summarize the diversity of immune cells in the TME, their impact on patient prognosis and treatment response, and the contributions of single‐cell RNA sequencing (scRNA‐seq) in understanding their functional heterogeneity.

**Methods:**

We analyzed recent studies utilizing scRNA‐seq to investigate immune cell populations in the TME, focusing on their interactions and regulatory mechanisms.

**Results:**

ScRNA‐seq reveals the functional heterogeneity of immune cells, enhances our understanding of their role in tumor antibody responses, and facilitates the construction of immune cell interaction networks. These insights provide guidance for the development of cancer immunotherapies and personalized treatment approaches.

**Conclusion:**

Applying scRNA‐seq to immune cell analysis in the TME offers a novel pathway for personalized cancer treatment. Despite its promise, several challenges remain, highlighting the need for further advancements to fully integrate scRNA‐seq into clinical applications.

## 1 Introduction

Tumor is a heterogeneous disease, with cancer initiation and progression consistent with changes in the surrounding stroma. Cancer cells can functionally shape their microenvironment by secreting various cytokines, chemokines, and other factors. This results in the reprogramming of surrounding cells, enabling them to play a decisive role in tumor survival and progression [1]. The tumor microenvironment (TME) is crucial in tumor development and heterogeneity [2]. The optimal phenotypic properties are determined by selective forces in the microenvironment, namely cellular characteristics that lead to maximum fitness [3]. In the TME, besides malignant cells, the composition and infiltration levels of immune cells vary across different tumor types [4]. When there is increased infiltration of T cells into the tumor tissue, the tumor size tends to remain smaller [5] resulting in a better prognosis for the patient [6]. Meanwhile, various other components within the tumor tissue, including macrophages and neutrophils [7], also play crucial roles in regulating the tumor immune microenvironment. Increasing evidence suggests that innate immune cells as well as adaptive immune cells in the TME promote tumor progression [1], which means that tumor development is closely regulated by immune cells in the TME. Furthermore, within the TME, interactions among all cell types via cellular communication mechanisms contribute to the complexity of tumor progression [8, 9, 10]. Recent studies using mass cytometry [11, 12] and bulk RNA‐seq analysis of immune cells residing in tumors [13] have offered comprehensive insights into the major immune cell subset composition. However, traditional research methods are difficult to fully reveal the diversity of immune cells and their role in tumor immune responses.

The concept and technical breakthrough of the scRNA‐seq method were first proposed by Tang et al. It opened a new way to expand the number of cells for the first time and promoted the study of the TME. Following this breakthrough, there has been a surge in the development of modifications and enhancements to scRNA‐seq technologies. To date, single‐cell RNA expression profiling is rapidly becoming an irreplaceable method in a variety of studies. Furthermore, leveraging information on gene expression, metabolites, intercellular communication, and spatial landscapes at both the mRNA and protein levels has the potential to address difficult questions of cellular composition and function in health and disease [14]. The development of scRNA‐seq has greatly facilitated the study of heterogeneous cell populations, which allows a comprehensive study of the transcriptome profile of a single‐cell population [15]. This technology has been used to study the TME in various cancer types. Through it, we can more comprehensively understand the diversity and heterogeneity of immune cells, as well as the cellular communication mechanisms, which are crucial for developing effective anti‐cancer immunotherapy strategies [8, 9, 10].

## 2 Application of scRNA‐Seq in Tumor Microenvironment

### 2.1 Applications of scRNA‐Seq Technology

Single‐cell genome sequencing is a new technology that amplifies and sequences the genome of individual cells, revealing differences and differentiation relationships among cell populations [16, 17]. Tissues and organs consist of cells in various states and functions, maintaining homeostasis in a complex microenvironment until extreme conditions potentially transform normal cells into tumors. To understand tumorigenesis, progression, metastasis, and treatment response, it is essential to study the TME, including immune and stromal infiltration [18]. ScRNA‐seq analysis can distinguish functionally healthy cells from cancer cells at different stages of tumor development. By identifying and determining sensitivities to different drugs, more precise prognosis and diagnosis can be achieved, and the most effective cancer treatment strategies can be developed. Various types of immune cells infiltrate the TME, namely T lymphocytes, CD8+ T cells [19, 20], tumor‐associated macrophages [21], and cancer‐associated fibroblasts (CAFs) [22, 23, 24, 25, 26, 27].

However, the immune response type and its influence on tumor growth, metastasis, and patient mortality differ greatly across various cancers and individual tumors [28]. The diversity and plasticity of immune cells enable dynamic responses to various immunogens. Despite advances in flow cytometry and bulk RNA‐seq, technical constraints hinder the thorough characterization of tumor‐infiltrating immune cells, limiting our understanding of malignant cells in most cancer patients. scRNA‐seq offers a powerful tool for analyzing the heterogeneity and interactions of these immune cells [29, 30, 31, 32, 33]. Through scRNA‐seq, the quantity and quality of tumor‐infiltrating immune cells have been resolved with unprecedented resolution for many cancers, including melanoma [23, 34, 35], lymphoma [36, 37, 38, 39, 40], glioma [41, 42, 43, 44], cholangiocarcinoma [44, 45, 46, 47, 48], nasopharyngeal carcinoma [49, 50, 51, 52], breast cancer [53, 54, 55, 56, 57], head and neck cancer [58, 59, 60, 61], colorectal cancer [62, 63, 64, 65, 66], gastric cancer [67, 68, 69, 70, 71, 72], liver cancer [21, 73, 74], kidney cancer [75, 76], pancreatic cancer [77, 78, 79, 80], bladder cancer [81, 82], ovarian cancer [83, 84, 85], and lung cancer [86, 87, 88]. These studies provide new insights into the composition, heterogeneity, dynamics, and regulation of immune cells in the TME.

In cancer research, scRNA‐seq technology can be used to identify or interrogate (1) rare subpopulations, (2) circulating tumor cells (CTCs), (3) tumor or immune microenvironment, (4) tumor heterogeneity and molecular subtypes, (5) mechanisms related to tumor occurrence, progression, metastasis, evolution, recurrence and drug resistance, and (6) cancer stem cells (CSCs) [89].

In digestive system tumors, single‐cell sequencing is extensively used to explore various aspects of gastric cancer. These include uncovering single‐cell lineage states, mapping TME dynamics, and identifying subtype‐specific expression programs [69], Additionally, it reveals the transcriptional heterogeneity associated with organ‐specific metastases in human gastric cancer [90], and highlights the prognostic significance of specific subpopulations, such as tumor‐associated fibroblasts, in predicting patient outcomes [91]. In colorectal cancer, scRNA‐seq has been extensively used to characterize the TME in colorectal cancer liver metastases [62] and to analyze the microenvironment in patients with varying treatment responses, such as those undergoing myeloid cell‐targeted therapy [63] or immunotherapy [64]. These studies have uncovered mechanisms underlying treatment resistance and highlighted the roles of unique cell subsets, including ANGPTL2+ tumor‐associated fibroblasts and SPP1+ macrophages, in promoting tumor metastasis [92]. Furthermore, scRNA‐seq identified two epithelial tumor cell states, leading to the proposal of two subtypes, iCMS2 and iCMS3, which refine the consensus molecular classification of colorectal cancer and provide critical guidance for clinical immunotherapy [65]. In liver cancer, scRNA‐seq has been used not only to map the fundamental TME [93] but also to analyze the immune landscape of hepatitis B virus‐related hepatocellular carcinoma, revealing its immunosuppressive features [74]. In addition, scRNA‐seq dissected the spatial heterogeneity and immune escape mechanisms of CTCs in hepatocellular carcinoma [94], as well as the impact of rare subpopulations, such as PPT1+ macrophages on immunotherapy [95]. And, the intratumoral heterogeneity of liver cancer was revealed through the analysis of liver cancer stemness‐related subpopulations [96]. scRNA‐seq data of the esophagus pointed out that our gastrointestinal metaplasia and Barrett's esophagus have the same original tumor and activated fibroblast microenvironment, which can be considered as molecularly similar entities in adjacent organs, paving the way for shared detection and treatment strategies [97]. It also pointed out that neoadjuvant therapy significantly regulates the cellular composition of the tumor immune microenvironment in esophageal cancer and proposed a potential model of the CCR4/CCR6 system to predict the benefits of combining neoadjuvant chemoradiotherapy with immunotherapy [98].

In lung cancer, in addition to scRNA‐seq analysis of tumor heterogeneity in the TME [99], comparative analysis of primary and metastatic TME [99] and analysis of the TME after neoadjuvant therapy [87] have also been performed. It is worth mentioning that some scholars have revealed the evolution caused by treatment of human lung cancer through scRNA‐seq [100]. In addition, scRNA‐seq of human and mouse lung cancer samples revealed the conservation of tumor‐infiltrating myeloid lineages, laying the foundation for future research on the potential of tumor‐infiltrating myeloid lineages as immunotherapy targets [101].

In breast cancer, scRNA‐seq has been used to map immune phenotypes in the TME [57]. It has also provided insights into the evolution of chemotherapy resistance in triple‐negative breast cancer (TNBC), revealing that drug‐resistant genotypes pre‐exist and are selected by neoadjuvant adaptive therapy in these patients [102]. Analysis of breast cancer‐infiltrating B lymphocytes showed that these cells exhibit mature and memory‐like characteristics, high clonality, enhanced class switching recombination, and somatic hypermutation, suggesting that B cell subsets may promote immune surveillance through multiple pathways [56]. Furthermore, scRNA‐seq revealed the cellular origins and evolutionary patterns of breast cancer in BRCA1 mutation carriers, identifying potential therapeutic targets for patients with BRCA1 mutations [103].

In urinary system tumors, scRNA‐seq has identified recurrence‐associated macrophages in renal tumors [76] and established that mesenchymal‐like tumor cells and myofibroblast CAFs are linked to the progression and immunotherapy response of clear cell renal cell carcinoma [104]. In bladder cancer, scRNA‐seq has provided detailed analyses of the immune landscape [105], elucidated mechanisms of cancer stem cell maintenance and epithelial–mesenchymal transition in recurrent cases [106] and identified an N‐cadherin 2‐expressing epithelial cell subpopulation that predicts responses to surgery, chemotherapy, and immunotherapy [107].

Integrating pan‐cancer scRNA‐seq data provides valuable insights for both basic and clinical research. For example, Wu et al. analyzed single‐cell transcriptomes of neutrophils across multiple cancer types [108]. They found that neutrophils' antigen presentation programs could be activated through leucine metabolism and subsequent histone H3K27ac modifications. These neutrophils, in turn, triggered both antigen‐specific and antigen‐independent T cell responses. By leveraging neutrophil delivery or dietary leucine interventions, researchers fine‐tuned immune responses to enhance the effectiveness of anti‐PD‐1 therapy in various mouse cancer models, offering critical guidance for advancing clinical immunotherapy.

ScRNA‐seq has also revealed the role of cuproptosis in the TME, providing new opportunities to investigate its contributions to cancer development and potential therapeutic targeting [109].

The application of single‐cell technology, combined with computational tools and publicly available datasets, is transforming drug discovery and development. Improved understanding of cell subtypes has opened new opportunities for target identification. For instance, the scDEAL algorithm integrates bulk RNA‐seq data associated with drugs into scRNA‐seq datasets, enabling predictive models to determine drug responses at the single‐cell level. Additionally, this approach identifies signature genes related to drug resistance, aiding studies on cell reprogramming, drug selection, and repurposing to enhance therapeutic outcomes [110].

**FIGURE 1:**
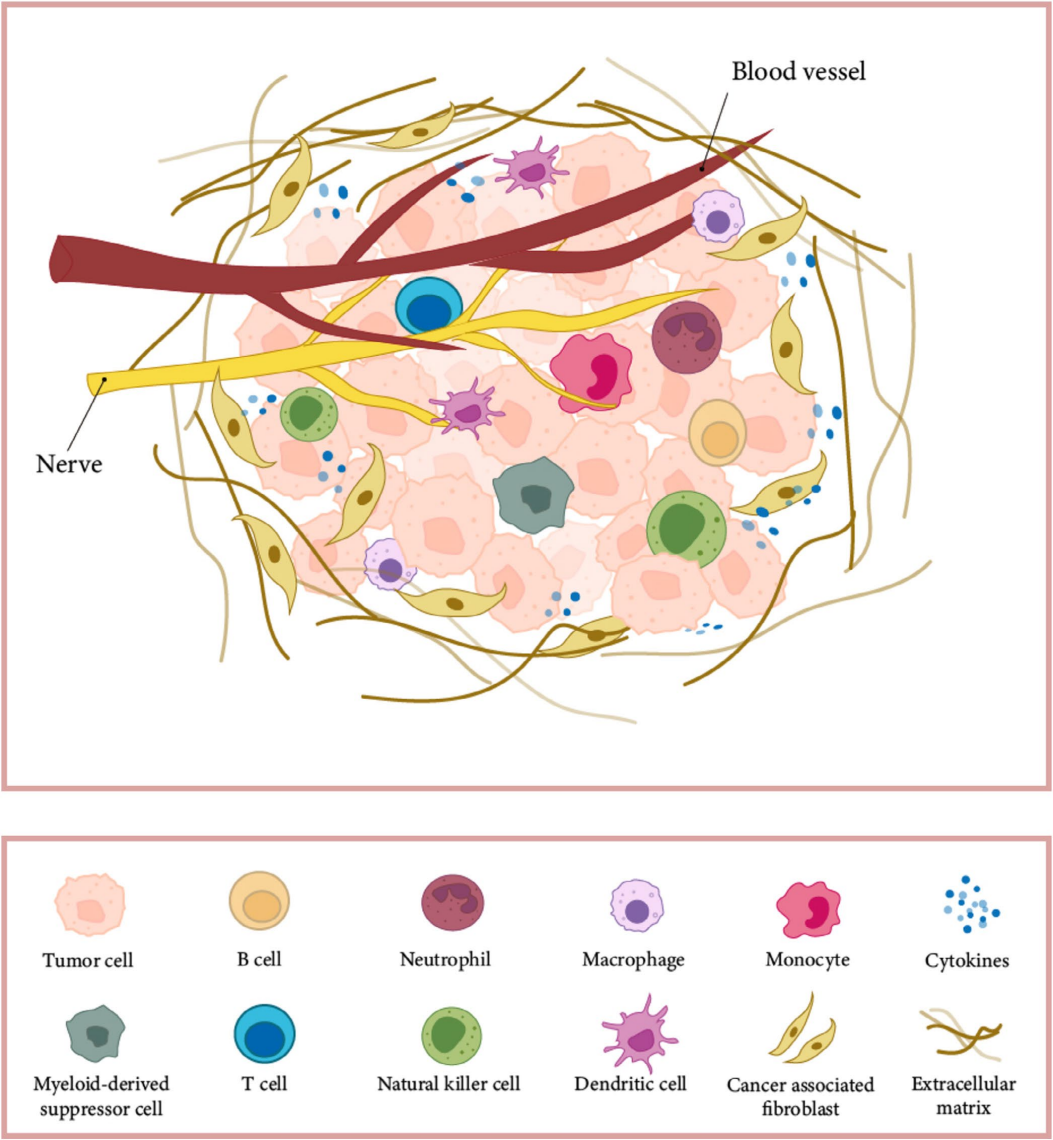
Tumor microenvironment.

ScRNA‐seq is also instrumental in selecting relevant preclinical disease models and uncovering new drug mechanisms of action. In clinical development, it facilitates better biomarker identification for patient stratification and enables precise monitoring of drug efficacy and disease progression [111]. Compared to traditional immunohistochemical immune scoring, single‐cell sequencing offers unprecedented resolution of immune cell infiltration in the TME. This capability allows for the identification of specific immune cell subpopulations that predict treatment response through surface markers, providing valuable guidance for precision tumor therapy and advancing personalized medicine (Figure 1).

### 2.2 Diversity of Immune Cells in the Tumor Microenvironment

ScRNA‐seq is becoming an invaluable tool for analyzing gene expression across thousands of cells simultaneously, facilitating detailed profiling of various cell types in tumors under different biological states and conditions [112]. Recent scRNA‐seq studies have revealed new insights into the diverse immune cell populations in the TME. For example, scRNA‐seq data show that there are five NK cell subgroups in healthy liver tissue. However, the transitional L3 subgroup with strong anti‐tumor activity and the CXCR6+CD16+L4 subgroup are absent in Hepatocellular carcinoma (HCC) and peri‐tumoral liver tissue [113]. as exemplified in a study focusing on pancreatic cancer [114]. The authors discovered spatially restricted enrichment of subpopulations of macrophages, dendritic cells, and cancer cells. In a study on gastric cancer scRNA‐seq data [90], it was found that the heterogeneity of immune and stromal cells within malignant epithelial subclusters creates a microenvironment conducive to tumor promotion and immune suppression. In addition, another scRNA‐seq data showed [115] that tumors from the same organ but from different sources also have immune cell diversity. In this study, the authors compared scRNA‐seq profiles of gliomas and lung–brain metastases. The results show that cells in the TME exist in heterogeneous subpopulations and that lung–brain metastases reprogram cells into an immunosuppressive state, including microglia, macrophages, endothelial cells, and CD8+ T cells, with unique cellular proportions and genetic traits.

Naturally, following the corresponding clinical treatment, the single‐cell landscape of tumor patients also undergoes reshaping. In a study [64], the authors investigated the dynamics of immune and stromal cells in colorectal cancer (CRC) patients undergoing PD‐1 blockade therapy. It was discovered that in tumors with complete pathological response, post‐treatment, the proportions of CD8+ Trm mitoses, CD4+ Tregs, and pro‐inflammatory IL1B+ Mono were consistently reduced, and the proportions of CD8+ Tem, CD4+ Th, and CD20+ B were increased. In tumors that respond or do not respond to immunotherapy, there is evident diversity among immune cells within the TME. According to a report by Magen et al., the response to immune checkpoint blockade (ICB) is associated with clonal expansion of intratumoral CXCL13+CH25H+IL‐21+PD‐1+CD4+ T helper cells (“CXCL13+ TH”) and granzyme K+ PD‐1+ effector‐like CD8+ T cells, while ultimately exhausted CD39^hi^TOX^hi^PD‐1^hi^CD8+ T cells predominate among nonresponders [116].

ScRNA‐seq has also revealed insights when applied to explore tumor heterogeneity. In sequencing datasets of lung cancer, it has been found that after targeted therapy, active T lymphocytes are present in residual lesions, while there is a decrease in macrophages. However, during disease progression, an immune‐suppressive cellular state exists, characterized by an increase in macrophages [100]. Immune cells in the TME exhibit significant differences between early and late‐stage cancers. A study employing scRNA‐seq analyzed tumors from patients with early or late‐stage high‐grade serous ovarian cancer and identified specific immune cell subgroups. For example, C7‐APOBEC3A M1 macrophages, CD8+ tissue resident memory, and tumor‐exhausted cells were preferentially enriched in early‐stage tumors [84].

ScRNA‐seq data also show [73] that, compared with primary liver cancer, the level of regulatory T cells in early recurrent liver cancer decreases, while dendritic cells (DC) increase and infiltrating CD8+ T cells increase. Furthermore, there is considerable heterogeneity among immune cells within the TME of primary and metastatic tumors [62, 117]. In a study of colorectal cancer liver metastasis [118], highly metabolically activated MRC1+CCL18+M2‐like macrophages were found at the metastasis site. In lung metastases, T cells exhibit exhaustion, while the normal resident bone marrow cell population is gradually replaced by monocyte‐derived macrophages and dendritic cells.

Aging is a major risk factor for cancer, with increasing incidence in the elderly linked to a decline in immune function. Exploring the heterogeneity and diversity of the TME in young and elderly patients is crucial for improving cancer prevention and treatment. A study using scRNA‐seq analyzed tumor‐infiltrating CD8+ T cells and identified novel age‐related T cell subsets. The findings revealed epigenetic differences between young and elderly T cells, uncovering molecular mechanisms of T cell dysfunction and highlighting the ability of tumors in the elderly to suppress immune responses [119]. In colorectal cancer, single‐cell sequencing showed that sporadic young patients have enriched B cells and naive T cells, while elderly patients have more effector T cells and plasma cells in the TME [120].

Gender differences also affect the TME. Studies have shown that male breast cancer patients have lower T cell infiltration and exhibit more immune dysfunction compared to female patients [121]. Different cancer subtypes also exhibit unique immune cell characteristics. In lung adenocarcinomas with EGFR mutations, CD1C+ dendritic cells are increased, and tumor‐associated macrophages display tumor‐promoting functions with significant heterogeneity [122]. In gliomas, animal models revealed that low‐grade gliomas (LGG) have increased infiltration of CD4+ T cells, CD8+ T cells, and NK cells, which is largely absent in high‐grade gliomas (HGG). Macrophages in LGG show an immunostimulatory phenotype, while those in HGG evolve into an immunosuppressive state [123].

ScRNA‐seq enables high‐resolution analysis of gene expression and cellular heterogeneity in the TME, uncovering diverse immune cell populations, dynamic therapeutic responses, and tumor progression mechanisms. It identifies unique subpopulations, such as absent NK cells in hepatocellular carcinoma or immunosuppressive states in brain metastases. Treatment‐induced changes, like PD‐1 blockade in colorectal cancer, reshape the TME by altering immune cell proportions. Tumor stage, age, gender, and subtype also influence TME composition, with specific immune cell profiles observed in early versus late‐stage cancers and between primary and metastatic sites. These insights highlight scRNA‐seq's role in understanding cancer biology, guiding immunotherapy, and tailoring personalized treatments (Table 1).

**TABLE 1:** Diversity of immune cells in the TME.

Tumor type	Sample type	Diversity of immune cells in the TME
Hepatocellular carcinoma (HCC) [113]	Hepatocellular carcinoma/peri‐tumoral liver tissue/healthy liver tissue	L3 subgroup with strong anti‐tumor activity and the CXCR6+CD16+L4 subgroup are absent in HCC and peri‐tumoral liver tissue
Pancreatic cancer [114]	Tumor/normal	Macrophages↑, dendritic cells↑
Gastric cancer [90]	Tumor/normal	Immune cells↑
Gliomas/lung–brain metastases [115]	Tumor (same organ)	In lung brain metastases, microglia, macrophages, endothelial cells, and CD8+ T cells, with unique cellular Proportions and genetic traits
Colorectal cancer [64]	Post‐treatment (complete pathological response (CPR)/non‐CPR	CD8+ Trm mitoses↓, CD4+ Tregs↓, and pro‐inflammatory IL1B+ Mono↓. CD8+ Tem↑, CD4+ Th↑, and CD20+ B ↑
HCC [116]	Respond or do not respond to immunotherapy	Respond: CXCL13+CH25H+IL‐21+PD‐1+CD4+ T helper cells ↑ (“CXCL13+ TH”) and granzyme K+ PD‐1+ effector‐like CD8+ T cells↑ Not respond: CD39hiTOXhiPD‐1hiCD8+ T cells
Lung cancer [100]	Targeted therapy response/disease progression	Response: active T lymphocytes↑, macrophages↓ Disease progression: macrophages↑
Ovarian cancer [84]	Early‐stage and late‐stage tumors	C7‐APOBEC3A M1 macrophages, CD8+ TRM, and TEX cells are preferentially enriched in early‐stage tumors
Liver cancer [62, 117]	Recurrent liver cancer/primary liver cancer	Regulatory T cells, dendritic cells (DC) ↑, infiltrating CD8+ T cells↑ in recurrent liver cancer
Colorectal cancer [118]	Liver metastasis/lung metastases	Metabolically activated MRC1+CCL18+M2‐like macrophages in liver metastasis. T cells exhibit exhaustion in lung metastases
Colorectal cancer [120]	Young/elderly patients	Young patients: B cells and naive T cells↑ Elderly patients: effector T cells and plasma cells↑
Breast cancer [121]	Male/female patients	Lower T cell infiltration and exhibit more immune dysfunction in male patients
Lung adenocarcinoma [122]	With EGFR mutations	CD1C+ dendritic cells↑, tumor‐associated macrophages↑
Glioma [123]	High‐grade/low‐grade	CD4+ and CD8+ T cells ↑and NK cells ↑ in low‐grade glioma

## 3 Immune Cell Functions Revealed by Single‐Cell Sequencing

### 3.1 Functional Heterogeneity of Immune Cells

ScRNA‐seq unveils the functional diversity of immune cells within the TME, delineating distinct functional characteristics among various cell subpopulations and their roles in tumor antibody responses. Presently, research on immune cell functionality predominantly centers on those pivotal in cancer immunotherapy. For instance, in 2021, the team led by Zemin Zhang constructed a pan‐cancer T cell atlas using scRNA‐seq data from 21 cancer types. They revealed diverse T cell composition patterns and inferred two primary developmental pathways of T cell exhaustion through computational analysis. These pathways involve effector memory T cells and tissue‐resident memory T cells, both prevalent across cancer types. Moreover, they unveiled transitions between terminal exhausted T cells and other cell types like NK‐like T cells, Tc17 cells, and CD8+ Treg cells, each exerting distinct functions within the tumor immune microenvironment [124].

In addition, according to scRNA‐seq data of patients treated with CART, CD8+ T cells are transformed into NK‐like T cells, resulting in the loss of the original function of CART cells [125]. However, there are reports that CART with a phenotype similar to NK has better efficacy [126]. Based on this analysis, subsequent clinical trials have proposed the predictive value of Ki67+ Treg in identifying immunotherapy beneficiaries and potentially guiding personalized treatment strategies [127]. Furthermore, in 2023, a study published in *Cell* [57] conducted an extensive scRNA‐seq analysis of NK cells from 24 different types of cancer. They observed heterogeneity in NK cell composition in a tumor‐type‐specific manner and identified a subset of tumor‐associated NK cells that are enriched within tumors, exhibiting impaired anti‐tumor function and correlating with poor prognosis and immunotherapy resistance. Specific subsets of bone marrow cells, particularly LAMP3+ dendritic cells, appear to mediate the regulation of NK cell anti‐tumor immunity.

There are also reports indicating that within the same type of tumor, NK cells exhibit functional heterogeneity. Professor Eric Vivier found through scRNA‐seq and analysis that NK cells in the bone marrow are heterogeneous under normal physiological conditions. Among them, a group of CD56bright NK0 cells are the precursors of NK1 and NK2 cells in peripheral blood. However, the heterogeneity of NK cells in the bone marrow of AML patients has changed significantly. Their NK cells downregulate the expression of CD160, and low expression of CD160 is associated with shorter survival of patients with acute myeloid leukemia [128]. It is worth mentioning that, for a specific subtype, NK cells may not necessarily play an immune protective role. There are reports that NKG2A+NK cells will cause immunotherapy failure. Combining anti‐NKG2A and anti‐PD‐L1 therapy can restore the complete response of immunotherapy in heterogeneous MHC‐I mouse models [129].

In the study of pancreatic cancer microenvironment, the exploration of tumor‐associated neutrophils (TAN) heterogeneity has found subpopulations associated with poor prognosis, including terminally differentiated tumor‐promoting subpopulations (TAN‐1), inflammatory subpopulations (TAN‐2), transitional subpopulations newly migrated to the TME (TAN‐3), and subpopulations, that preferentially express interferon‐stimulated genes (TAN‐4) [130]. In their report on gastric cancer metastasis, Qian et al. proposed that the inhibition of neutrophil polarization‐related genes (such as LCN2) was found through scRNA‐seq to contribute to the lymph node metastasis of gastric cancer [117]. Subsequent reports have also pointed out that the LCN2/24p3R/JNK/c‐Jun/SPARC axis is crucial in the malignant progression of GC, providing new prognostic markers and therapeutic targets [131]. In addition, TANs have a pre‐tumor phenotype and immunosuppressive ability, and PD‐L1+ TANs inhibit T cell cytotoxicity [132].

Identifying specific functional immune cell subtypes in the TME by scRNA‐seq will also aid cancer immunotherapy and further exploration. Sathe et al. [133] conducted a study to investigate cellular changes in the TME of colorectal cancer metastases in the liver. They discovered specific SPP1+ macrophages characterized by altered metabolism and foam cell characteristics, as well as increased extracellular matrix tissue activity. SPP1+ macrophages and fibroblasts express complementary ligand‐receptor pairs, suggesting that their gene expression programs may influence each other. The intercellular network between SPP1+ macrophages and fibroblasts supports the growth of colorectal cancer in the liver immunosuppressive metastatic microenvironment, providing a potential target for immune checkpoint‐resistant microsatellite stable tumors. Corresponding studies also pointed out that SPP1+ macrophages are metastasis accelerators of colorectal cancer [92] and are associated with cell senescence, leading to poor prognosis in tumor patients [134]. In addition, pan‐cancer single‐cell sequencing analysis revealed that AtM B cells can impair the number and function of T cells, thereby promoting the formation of an immunosuppressive microenvironment. In clinical cohorts, AtM B cells are significantly associated with immunotherapy resistance and poor prognosis, laying the theoretical foundation for the development of new immunotherapy methods targeting B cells [135]. In addition, pan‐cancer scRNA‐seq data also showed that patients with high expression of tumor‐associated B cell (TAABs) characteristics usually have a better response to immunotherapy [136].

### 3.2 Immune Cell Interaction Network

By analyzing the gene expression of individual immune cells, we can construct networks that illustrate interactions between immune cells in the TME, offering deeper insights into the regulatory mechanisms governing immune cell dynamics. Tumors and their surrounding microenvironments form complex cellular communities capable of modulating the function of local immune cells.

Investigating the interaction networks within these communities reveals communication dynamics that shape these ecosystems [21, 48, 58, 137, 138, 139] and provides valuable guidance for the development of effective cancer immunotherapies [140, 141]. For instance, cell communication analysis using scRNA‐seq data has identified changes in receptor–ligand responses to anti‐PD‐1 (aPD‐1) therapy across all immune cells. Several interactions were found to be downregulated, including SPP1‐CD44, which mediates macrophage polarization and promotes immune escape; GAS6‐AXL; and TGFβ‐TGFBR1/TGFBR2, both associated with an immunosuppressive TME. This analysis also highlighted the transformation of ligand–receptor interactions following a PD‐1 treatment, indicating an immune‐stimulatory state and identifying potential targets for combination therapies [142].

Communication between different cell types is closely linked to key mechanisms in tumorigenesis, tumor evolution, therapeutic resistance, immune infiltration, and tumor prognosis [143]. Understanding these interactions can uncover critical pathways and improve therapeutic strategies. Studies have shown that *Helicobacter pylori* infection enhances the number and intensity of intercellular communication and activates signaling pathways, such as TNF‐TNFR1 through multicellular communication, which may play a key role in the development of *H. pylori* ‐related gastric cancer. Using CellPhoneDB28 to analyze cell communication by searching for ligand–receptor interactions between malignant cells (ligand source) and T cells (receptor source), it was revealed that SPP1‐CD44 is the top interaction pair between malignant cells and T cells, further supporting the key role of SPP1 in the tumor ecosystem and suggesting that the TME is polarized as tumor branches evolve [89, 144]. In addition, in a cell communication analysis of scRNA‐seq data of the dynamic evolution from precancerous lesions to invasive lung adenocarcinoma, it was shown that no communication between cancer cells and TME cells through TGF‐β signaling was observed in adenocarcinoma in situ and microinvasive adenocarcinoma, but this interaction was significantly enhanced in invasive adenocarcinoma, especially in NK, hypertrophic and MALT B cells. This suggests that in the early stage, TGF‐β inhibits cell growth as a tumor suppressor pathway, while in the later stage, TGF‐β promotes invasion and metastasis [145]. Li et al. revealed the development mechanism of actinic keratosis to skin squamous cell carcinoma through single‐cell transcriptome sequencing, and proposed through cell communication that as the malignancy of skin squamous cell carcinoma increases, the interaction between cells is significantly enhanced. Signaling pathways that play a key role in tumor progression, including MHC‐II [146], Laminin [147], and TNF [148], provide important instructions for tumor prevention and treatment [149].

ScRNA‐seq can reveal the TME during treatment resistance through cell‐to‐cell interactions, providing a potential solution for tumor treatment resistance. For example, scRNA‐seq of gastric cancer resistance revealed complex interactions between apical membrane cells and resident macrophages and related molecular mechanisms of cell interaction. Ligand–receptor analysis revealed unique interaction molecular patterns in the treatment response group and the resistance group, providing valuable insights into the potential mechanisms affecting treatment outcomes [150]. A cell communication analysis of imatinib resistance showed that in the imatinib‐resistant TME, tumor cells with activated immunity and cytokine‐mediated immune responses interacted with a higher proportion of Treg cells through the TIGIT‐NECTIN2 axis. Future immunotherapy strategies targeting Treg may provide new directions for the treatment of imatinib‐resistant patients [151]. In a scRNA‐seq study of lung adenocarcinoma, the authors found that mast cells are an important source of CCL2 and are associated with the recruitment of CCR2+CTL. Increased infiltration of mast cells and CCR2+CTL and their co‐localization are closely associated with a good prognosis after surgery but are not associated with improved survival after chemotherapy. Overall, these findings suggest that targeting mast cells may be an immunotherapy strategy for lung adenocarcinoma [152].

Cell–cell communication analysis has highlighted the critical role of immune cell interactions in modulating immune cell infiltration and function within the TME. For example, studies have shown that LCK in melanoma subpopulations interacts with CD8 receptors on T cells to promote immune activation [153]. In HBV‐related hepatocellular carcinoma, tumor‐associated macrophages were found to suppress T cell infiltration into tumors [74]. ScRNA‐seq data have also revealed the distinct immunosuppressive microenvironment of TNBC compared to HER2+ or luminal breast cancers. Cell–cell communication analysis demonstrated that these immunosuppressive traits are driven by enhanced T cell‐B cell interactions in TNBC. Tregs and exhausted CD8+ T cells in this setting exhibit increased immunosuppressive features and higher dysfunction scores [154].

Utilizing scRNA‐seq to investigate immune cell‐tumor cell interactions has proven to be a powerful approach for prognosis prediction. For instance, CellChat analysis identified SPP1‐CD44 interactions between tumor cells and T cells, with SPP1 expression in malignant cells negatively correlating with T cell proliferation scores. This finding confirmed SPP1 as a prognostic marker for liver cancer [155]. In recurrent liver cancer, scRNA‐seq revealed that interactions between bone marrow‐derived cells and T cells mediate T cell exhaustion and immunosuppression. Newly diagnosed recurrent liver cancer patients demonstrated a stronger response to PD‐1 immunotherapy, highlighting the significance of these interactions [156]. Additionally, in a mouse model of B cell lymphoma, it was observed that CAR‐T cells rely on cytokine‐mediated crosstalk with immune cells in the TME to achieve optimal activity, leading to improved prognoses [157].

These studies emphasize the importance of immune cell interactions and immune‐tumor cell crosstalk as predictors of therapeutic efficacy and prognosis. Understanding these interactions provides precise diagnostic and therapeutic targets and supports the development of more effective immunotherapies. A deeper exploration of the diversity of cellular interactions within the TME and their impact on treatment outcomes is essential for optimizing patient classification and identifying new intervention strategies.

## 4 A New Perspective on Personalized Treatment

### 4.1 Personalized Treatments Through Single‐Cell Sequencing

By analyzing the sequencing results of the TME, we can provide personalized treatment plans for each patient, targeting the specificity of immune cells. Single‐cell RNA sequencing (scRNA‐seq) revealed significantly enriched transcriptional characteristics of FCRL4+FCRL5+ memory B cells and CD16+CX3CR1+ monocytes in patients demonstrating major pathological responses, serving as predictive factors for immunotherapy response [87]. In addition, some scholars have mapped the transcriptome of cancer cells at single‐cell resolution as well as the tumor heterogeneity and TME in recurrent drug‐resistant glioblastoma multiforme and found that it shows a reduced proportion of microglia, providing new insights into therapeutic strategies for recurrent glioblastoma [158].

ICB therapy has revolutionized cancer treatment. While predictive biomarkers and strategies to enhance clinical response have mainly focused on T cells [159], other immune subsets may also contribute to anti‐tumor immunity [160, 161, 162, 163, 164]. A previous trial of neoadjuvant ICB in melanoma patients showed targeted expression profiling [165] that B cells may play this role in anti‐tumor immunity.

Research utilizing scRNA‐seq has validated how different routes of administration of anti‐tumor vaccines alter bone marrow cells in the TME. It was found that in the TME of vaccination methods leading to tumor regression, there is a reduction in the expression of immunomodulatory genes (Chil3, Anxa2, Wfdc17) in intratumoral monocytes. In humans, the gene features of Chil3+ monocytes are enriched in CD16‐ monocytes and are associated with poorer outcomes [166]. This provides new ideas for more precise and effective vaccine treatment of tumors.

A scRNA‐seq study on tumor‐infiltrating B cells in breast cancer revealed five clusters of TIL‐B and identified follicular B cells closely associated with the efficacy of immunotherapy, explaining how B cells significantly promote anti‐tumor immunity at both the single‐cell and clinical levels. Another scRNA‐seq study on B cells in colorectal cancer with liver metastasis also identified a B cell subtype, immature plasma cell cluster α, highly associated with metastasis. Mechanistically, inhibiting the Wnt and TGF‐β pathways in cancer cells can promote activated B cells to migrate via the SDF‐1‐CXCR4 axis. This study provides a basis for subsequent drug development and treatment of colorectal cancer liver metastasis [167]. Lei [168] and others used scRNA‐seq to propose that mCAF constitutes an invasive CAF subset, promotes tumor cell invasion, activates endothelial cells to trigger angiogenesis, and cooperates with SPP1+ tumor‐associated macrophages to induce tumor progression, ultimately reducing hypopharyngeal squamation. Proposing a new anti‐hypopharyngeal squamous cell carcinoma treatment strategy that inhibits mCAF activation. Another scRNA‐seq data on cutaneous T‐cell lymphoma [39] showed that increased expression of S100A9 and its receptor TLR4, as well as activation of downstream Toll‐like receptors and NF‐κB pathways, were observed. In response to this result, the authors used taquinimod to inactivate the NF‐κB pathway, which provides a basis for the treatment of cutaneous T‐cell lymphoma. Therapeutics provide new targets and potential therapeutic strategies. In a study on liver metastasis of colorectal cancer caused by oxaliplatin treatment, the authors used scRNA‐seq to study the pre‐metastasis niche and found that macrophages showed strong inhibition after oxaliplatin treatment. The ability of T cells to activate. Further studies showed that the number of T cells in the liver was significantly reduced, especially CD8+ T cells, with reduced proliferation, activation, and killing capabilities. When mice received T cell supplementation therapy, oxaliplatin‐induced metastasis was significantly eliminated, indicating that the liver microenvironment induced by oxaliplatin can be reversed by infusion of T cells, providing evidence for the development of colorectal cancer after oxaliplatin chemotherapy. New ideas have emerged for the treatment of liver metastases [169].

Research utilizing scRNA‐seq has also revealed the evolution of immune cells in the TME induced by cancer therapy. It has been noted that during the process of targeting residual lesions, there is an induction of a more inflammatory phenotype characterized by reduced infiltration of T cells and immunosuppressive macrophages. Conversely, in progressive disease, there is an enrichment of macrophages expressing IDO1, regulatory T cells, and other immunosuppressive T cell populations, all of which are unfavorable for establishing an effective immune response [100]. In terms of directly utilizing immune cells for therapeutic purposes, scRNA‐seq also provides corresponding guidance. The use of scRNA‐seq to map TIL‐B clonotypes to phenotypes has created a new era, that is, from the TIL‐B phenotype of interest. Recombinant antibodies were generated and used to probe antigen specificity and effector mechanisms at an unprecedented scale [170]. scRNA‐seq is used to guide CAR‐T development and identify target antigens, which also provides new ideas for personalized treatment. One study identified potential CAR‐T cell targets, such as CSF1R and CD86 [171]. These targets have shown strong efficacy with minimal off‐target toxicity, supporting their clinical development. In addition, research has also used scRNA‐seq to design simulated memory killer cells and “speedingCars” for CAR‐T cell development [172] (Table 2).

**TABLE 2:** Personalized treatment strategies.

Tumor type	Sample type	Personalized treatment strategies
Non‐small cell lung cancer [86]	Lung cancer with major pathological reactions	FCRL4+FCRL5+ memory B cells and CD16+CX3CR1+ monocytes in patients showing major pathological responses, serving as predictive factors for immunotherapy response
Glioblastoma [158]	Relapsed drug‐resistant glioblastoma multiforme	Recurrent glioblastoma multiforme Glioblastoma tissue shows a reduced proportion of microglia, providing new insights into therapeutic strategies for recurrent glioblastoma
Melanoma [165]	Respond or do not respond to ICB	B cells may also contribute to anti‐tumor immunity
Colorectal cancer [167]	Primary/metastatic	This study elucidates the preventive role of modulating B cell subtypes in CRC, potentially providing a basis for subsequent drug development and management of colorectal cancer liver metastasis
Hypopharyngeal squamous cell carcinoma [168]	Tumor/normal	A new anti‐hypopharyngeal squamous cell carcinoma treatment strategy that inhibits mCAF activation
Cutaneous T‐cell lymphoma [39]	Lymphoma	Taquinimod was used to block the S100A9‐TLR4 interaction to inactivate the NF‐κB pathway, inhibit the growth of cutaneous T‐cell lymphoma tumor cells, and trigger cell apoptosis
Colorectal cancer [169]	Colorectal cancer liver metastases	The liver microenvironment induced by oxaliplatin can be reversed by infusion of T cells, providing evidence for the development of colorectal cancer after oxaliplatin chemotherapy New ideas have emerged for the treatment of liver metastases
Lung cancer [100]	Before and during targeted therapy	Inducing a more immunostimulatory phenotype during targeted therapy
Acute myeloid leukemia [171]	AML/normal	Identified potential CAR‐T cell targets, such as CSF1R and CD86

### 4.2 Challenges and Prospects

The primary approach to cancer treatment today is multimodal therapy, which includes surgery, chemotherapy, immunotherapy, and radiation therapy. While these treatments are effective for some patients, they offer limited clinical benefits for many others and can lead to treatment‐related morbidity. Furthermore, various clinical outcomes, such as treatment resistance, recurrence, and toxicity, are attributed to the intratumoral heterogeneity.

In clinical settings, tumor heterogeneity poses challenges such as hindering early response to resistant subclones and affecting the accurate quantification of driver cells and CSCs. Traditional bulk sequencing techniques struggle to quantify these features, as the genomic characteristics of rare cell populations are masked, leading to inaccurate inferences about tumor cell composition.

As mentioned earlier, scRNA‐seq technology plays a crucial role in predicting patient prognosis, treatment response, and exploring mechanisms of immunotherapy resistance. Analyzing the heterogeneity of immune cells in the TME and the corresponding pathways and molecular markers of cell interactions is essential for assessing the efficacy of cancer treatments within complex cellular ecosystems. Additionally, combining scRNA‐seq with multi‐omics will provide a more comprehensive understanding of cell types and states. Integrating single‐cell transcriptomics and proteomics data helps to understand how transcriptomic states transform into functional phenotypes and the underlying heterogeneity at transcriptional and translational levels, leading to a deeper understanding of tumor evolution. Integrating live‐cell imaging data with scRNA‐seq can analyze more complex cell phenotypes and their spatial localization and states [173].

However, applying scRNA‐seq technology to the clinic, which involves analyzing the immune cell status and heterogeneity in the TME of each patient, requires strict sample collection and preservation capabilities, as well as expertise in clinical bioinformatics analysis. Moreover, it is subject to limitations, such as high sample quality requirements, limited throughput, inevitable technical errors, and high costs [174], there is still a long way to go before this technology can be applied to clinical applications on a large scale and guide clinical diagnosis and treatment.

To gain a fundamental understanding of the molecular hierarchy from the genome to a phenomenon in single cells, multi‐omics approaches with single‐cell and spatial resolution are necessary. They enable investigation of the molecular dynamics between gene regulation at the epigenome level and gene expression at the transcriptome and/or proteome level [175]. As a recently developed technology, scRNA‐seq still has some limitations. For example, positional information is not preserved when measuring cell states using scRNA‐seq [15].

Spatial transcriptomics (ST) is a new biotechnology that allows visualization and quantitative analysis of transcriptomes at spatial resolution in tissue sections to find spatial interactions between cells, thus compensating for the lack of spatial information in scRNA‐seq, which results in the loss of critical spatial context. Combining ST and scRNA‐seq helps overcome the limitations of scRNA‐seq (lack of spatial information) and ST (not at single‐cell resolution) [176].

Multimodal intersection analysis is one of the useful methods [177] that provides meaningful biological insights for research by combining ST and scRNA‐seq. In addition, high‐throughput multi‐omics technologies, such as genomics, epigenomics, transcriptomics, proteomics, and metabolomics, have also facilitated the mapping of different molecular layers, greatly expanding the scope of biological analysis and our understanding of complex biological systems. For example, the combination of scRNA‐seq and DNA sequencing can not only provide us with more information about the interaction between the epigenome and transcriptome but also provide a deep understanding of the transcriptomic phenotype of shared somatic mutations in DNA [112].

**FIGURE 2:**
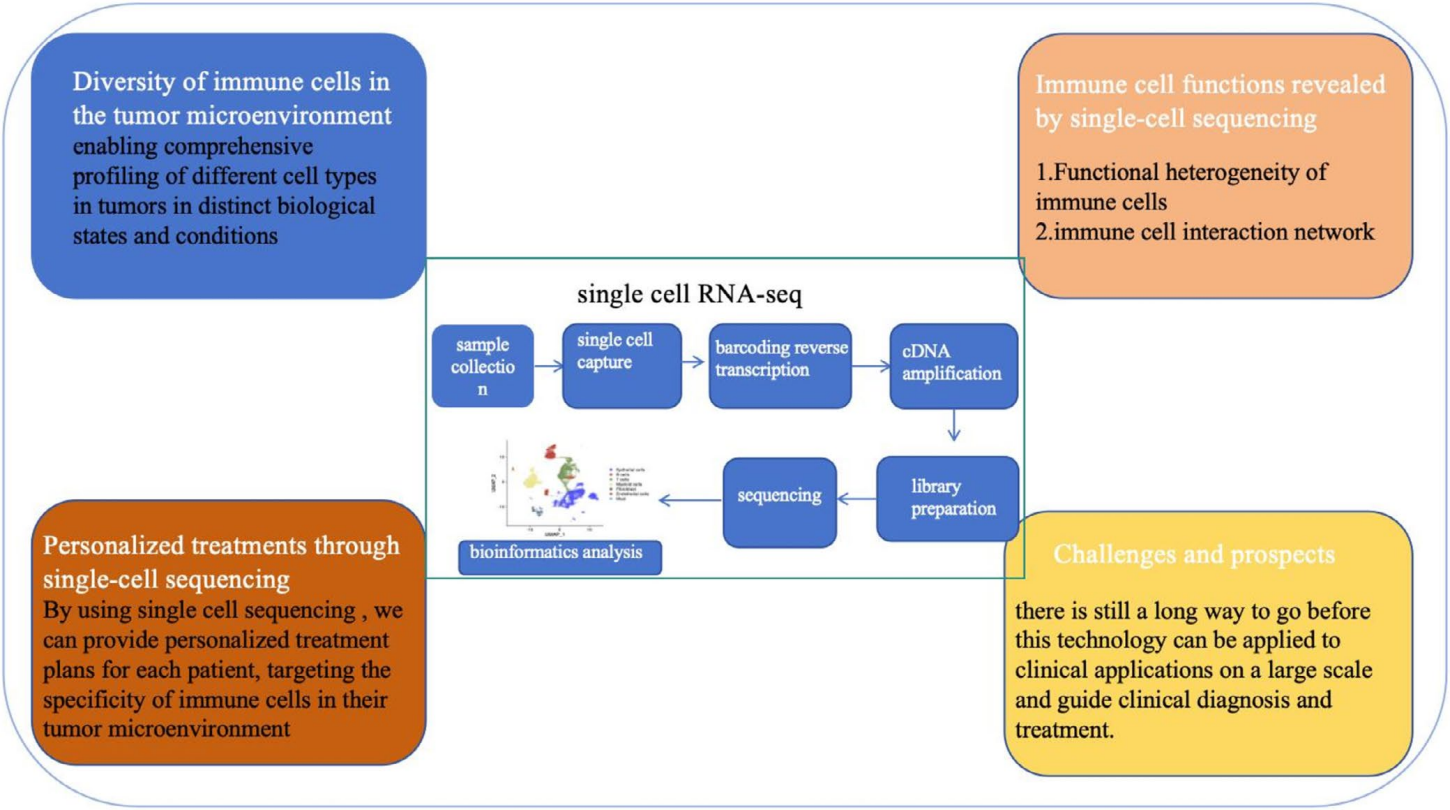
Challenges and prospects.

Metabolites play a vital role in various cellular activities, such as cell signaling, energy transfer, and intercellular communication. By combining spatial dynamic metabolomics with scRNA‐seq to characterize dynamic cellular metabolism associated with phenotypic and transcriptomic features, a metabolic map of the tumor is provided. It can be used for early detection of tumors [178], progression [179], etc. (Figure 2).

## 5 Conclusion

Through scRNA‐seq technology, we can gain a more comprehensive and in‐depth understanding of the diversity and functional heterogeneity of immune cells in the TME. Immune cells in tumors exhibit significant heterogeneity across different tumor sites, primary and metastatic tumors at the same site, and tumors subjected to different treatment regimens. This heterogeneity plays a crucial role in analyzing and predicting the prognosis of cancer patients, as well as understanding the mechanisms of treatment response and drug resistance. Additionally, the precise detection and targeted treatment of a patient's TME through scRNA‐seq analysis of immune cell interactions open a new era of precision medicine, providing valuable guidance for more precise and effective immunotherapy.

However, there are still many technical and cost‐related challenges to be addressed before scRNA‐seq can be applied clinically for personalized diagnosis and treatment. Nonetheless, if scRNA‐seq can be utilized to probe the immune microenvironment of each tumor patient to guide specific treatments, it will be a major milestone in cancer therapy.

## Author Contributions

**Lujuan Ma:** conceptualization (equal), investigation (equal), methodology (equal), writing – original draft (equal), writing – review and editing (equal). **Yu Luan:** conceptualization (equal), investigation (equal), methodology (equal), writing – original draft (equal), writing – review and editing (equal). **Lin Lu:** funding acquisition (equal), project administration (equal), supervision (equal).

## Conflicts of Interest

The authors declare no conflicts of interest.

## Data Availability

The authors have nothing to report.
